# *Coriolus Versicolor* and *Ganoderma Lucidum* Related Natural Products as an Adjunct Therapy for Cancers: A Systematic Review and Meta-Analysis of Randomized Controlled Trials

**DOI:** 10.3389/fphar.2019.00703

**Published:** 2019-07-03

**Authors:** Linda Zhong, Peijing Yan, Wai Ching Lam, Liang Yao, Zhaoxiang Bian

**Affiliations:** ^1^Hong Kong Chinese Medicine Clinical Study Centre, School of Chinese Medicine, Hong Kong Baptist University, Hong Kong; ^2^Institution of Clinical Research and Evidence Based Medicine, Gansu Provincial Hospital, Lanzhou, China

**Keywords:** Coriolus versicolor, Ganoderma lucidum, natural products, cancer therapies, systematic review, meta-analysis

## Abstract

**Background:** Cancer incidence and mortality rates keep rising globally. *Coriolus versicolor* and *Ganoderma lucidum* related natural products are commonly applied as a complementary therapeutic option for different stages and types of cancers. The aim of this study is to evaluate the efficacy and safety of the products for cancer therapy.

**Methods:** Randomized controlled trials were identified by systematic search over seven databases from inceptions to May 10, 2019. Two independent reviewers extracted data and assessed the study quality. Meta-analyses were performed to pool hazard ratio (*HR*), risk ratio (*RR*), mean differences (*MD*), and 95% *CI* using random-effects models. The sources of heterogeneity were explored by subgroup analyses and sensitivity analyses. Publication bias was detected by Funnel plots, Begg’s test, and Egger’s test.

**Results:** Twenty-three trials involving 4,246 cancer patients were included in this work. *C. versicolor* and *G. lucidum* related natural products were significantly associated with lower risks of mortality (*HR*: 0.82; 95% *CI*: 0.72, 0.94) and higher total efficacy (*RR*: 1.30; 95% *CI*: 1.09, 1.55), but not associated with control rate (*RR*: 1.05; 95% *CI*: 0.96, 1.14) compared with control treatment. There was no significant difference between *C. versicolor* related natural products and control treatment in the effect on relapse-free survival (*HR*: 1.19; 95% *CI*: 0.91, 1.55). Compared with control treatment, *C. versicolor* and *G. lucidum* related natural products had a favorable effect on elevated levels of CD3 (*MD*: 9.03%; 95% *CI*: 2.10, 16.50) and CD4 (*MD*: 9.2%; 95% *CI*: 1.01, 17.39), but had no effect on the levels of CD8 (*MD*: −5.52%; 95% *CI*: −23.17, 12.13), CD4/CD8 (*MD*: 0.73; 95% *CI*:-0.45, 1.91), or NK(*MD*: 5.87%; 95% *CI*: −1.06, 12.8).

**Conclusion:** In this meta-analysis, we found that *C. versicolor* and *G. lucidum* related natural products might have potential benefits on the overall survival and quality of life in cancer patients.

## Introduction

The burden of cancer continues to increase globally. According to WHO statistics, cancer is the second leading cause of death, accounting for 8.8 million deaths in 2015 (Organization, 2018). Anticancer therapies, for instance, surgery, chemotherapy, radiotherapy, and targeted cancer immunotherapy, are examples on controlling cancer cell growth, prolonging survival time, and improving quality of life to some extent. However, these therapies either alone or in combination have been shown to have various limitations and can result in severe side effects, which include an increased risk of subsequent cancers and lowered quality of life that vary with clinical factors (e.g., cancer type and treatment) and patient characteristics (e.g., age, sex, and comorbidity) (Hodge et al., 2012; Zigler et al., 2013; Miller et al., 2016).

In the past few decades, *Coriolus versicolor* (taxonomic name, *Trametes versicolor*; Chinese name; *Yun Zhi*) and its related mushrooms recorded in traditional Chinese medicine (TCM) literature have found their way to the market in Asian countries as anticancer remedies, and potentially play an important role in the whole course of cancer treatment such as the recovery stage of post-operation, and the stages during and after radiotherapy or chemotherapy (Sanodiya et al., 2009; Zhou et al., 2014). However, they have long been clinically confused based on their similar appearance and nature of medicinals according to the TCM theory (Pharmacopoeia, 2015). *In vitro* studies suggested that both *C. versicolor* (*Yun Zhi*) and *Ganoderma lucidum* (*Ling Zhi*) extracts, for instance, polysaccharide krestin (PSK), polysaccharide peptide (PSP) in *C. versicolor*, and beta-glucans, triterpenes in *G. lucidum*, possess selective cytotoxic activity against certain tumor cells (Yang et al., 1992; Dong et al., 1997; Jin et al., 2012). They may also activate various types of immune effector cells to enhance their anticancer activity, for instance, B lymphocytes, T lymphocytes, cytotoxic T cells, natural killer cells, and lymphokine activated killer cells (Xu et al., 2011; Yousefi et al., 2017). Furthermore, significant reduction of the tumor size after prolonged administration with the extracts was clearly shown in mice and the extract appeared to be effective for the prophylaxis against cancers (Tsukagoshi et al., 1984; Ren et al., 2012; Chen et al., 2014).

Compared to the supporting evidence from laboratory and animal tests, human trial of *Yun Zhi* and *Ling Zhi* extracts is just having its start. In the past 40 years, trials were mainly conducted on patients in Asia with breast cancer, colorectal cancer, gastric cancer, and non-small cell lung cancer, etc. and the trial data were scattered in regional databases (Torisu et al., 1990; Nakazato et al., 1994; Tsang et al., 2003; Kuo et al., 2012). Also, there was no systematic review to integrate the outcome measurements in different trials to form strong evidence. As a result, in order to provide better understanding of their clinical effect for physicians and other health care providers, we summarized trial results using *C. versicolor* and *G. lucidum* related natural products as adjuvant cancer treatment in different stages and kinds of cancer lesions from various databases. We hope this review can contribute a comprehensive view of current existing evidence to facilitate development of more effective natural products for public good.

## Methods

### Search Strategy

The systematic review was performed in accordance with the Preferred Reporting Items for Systematic Reviews and Meta-Analyses (PRISMA) guidelines (Moher et al., 2009). We searched PubMed, Embase, Cochrane Library, Web of Science, China National Knowledge Infrastructure (CNKI), Wanfang Database, and Chinese Scientific Journal Database (VIP) from inceptions to May 10, 2019, and identified randomized controlled trials with *C. versicolor* and *G. lucidum* related natural products for cancer patients. The search strategy was conducted by medical subject headings with text words. We referred to the published Cochrane protocol about *C. versicolor* and *G. lucidum* to initiate our search strategies (Pilkington et al., 2016). We also consulted all the searching names with the Chinese Medicine pharmacists in mainland China and Hong Kong. The completed search terms about *C. versicolor* and *G. lucidum* included as follows: *C. versicolor*, *T. versicolor*, *Polyporus* *versicolor*, *Polystictus versicolor*, Kawaratake, *Yun Zhi*, *Ling Zhi*, polysaccharide-K, PSK, krestin, polysaccharopeptide, polysaccharide-peptide, PSP, VPS, turkey tail, cloud mushroom, and unji mushroom.

In addition, the reference lists of the included studies were also checked, so as to supplement possible relevant literatures.

### Study Selection

Two reviewers independently screened and selected the searched articles according to the inclusion criteria (LZ and PY): 1) patients with cancer confirmed by pathology; 2) *C. versicolor* and *G. lucidum* related natural products were used as an intervention alone or combined with other drugs, without limitation on drug regimen, dosage and, course of treatment; and 3) must be randomized controlled trials. The following articles were excluded: 1) case series or reviews and conference abstracts; 2) valid original data were unable to obtain even when contacting the author; and 3) similar studies were reported without additional data to analyze and extract.

### Data Extraction

Two reviewers (LZ and PY) independently extracted data on participant characteristics from the selected studies in a standardized data extraction form. We extracted the following information from each included article: first author, year of publication, country, the type of tumor, number of participants, participant characteristics, the characteristics of the products (the type, dose, start time, duration of therapy), mean follow-up duration, number of dropout, controlled intervention, and outcome data.

### Definition of Outcomes

We included three primary outcomes to compare the effectiveness and safety of *C. versicolor* and *G. lucidum* related natural products for cancer in the analysis: 1) overall survival (OS); 2) relapse-free survival (RFS) rate; and 3) clinical efficacy. OS and RFS are defined by the individual study. Clinical efficacy was evaluated by investigators using Macdonald criteria (Macdonald et al., 1990). There were four “response” categories: complete response (CR), partial response (PR), stable disease (SD), and progressive disease (PD). Total efficacy means CR+PR; control rate means CR+PR+SD.

The secondary outcomes recorded were 1) immune-modulating effects including cluster of differentiation 3 (CD3); cluster of differentiation 4 (CD4); cluster of differentiation 8 (CD8); CD4/CD8; and natural killer cell (NK); 2) the post-treatment Karnofsky Performance Status (KPS) Score Change; and 3) adverse events. The post-treatment KPS score change was divided into obvious effectiveness (had an improvement of more than 20 points in the KPS score), effectiveness (an improvement of more than 10 points), stabilization (an improvement of less than 10 points or had no change), and invalid (a decrease in KPS score). Total effectiveness means obvious effectiveness plus effectiveness (Zhou and Lin, 2003).

### Risk of Bias Assessment

Two review authors (LZ and PY) assessed potential risks of bias for all included studies using the Cochrane’s tool for assessing risk of bias (Higgins JPT and Sterne, 2011). The tool assesses bias in six different domains: sequence generation; allocation concealment; blinding of participants, personnel, and outcome assessors; incomplete outcome data; selective outcome reporting; and other sources of bias. Each domain receives a score of high, low, or unclear depending on each review author’s judgment. A third review author acted as an adjudicator in the event of disagreement. Where doubt existed as to a potential risk of bias, we contacted the study authors for clarification.

### Statistical Analysis

In this meta-analysis, risk ratio (*RR*) and 95% confidence interval (*CI*) were considered as the effect size for dichotomous outcomes; weighted mean differences (WMD) with 95% *CI* were calculated as the effect size for continuous outcomes. For time-to-event data, we will pool hazard ratio (*HR*). Forest plots were produced to visually assess the effect size and corresponding 95% *CI* using random-effects models. Heterogeneity between studies was assessed *via* the forest plot, while *I* *^2^* values described the total variation between studies. *I* *^2^* values of <25%, 25–50%, and >50% indicated low, moderate, and high heterogeneity, respectively. Subgroup analyses were used to explore and interpret the sources of heterogeneity; to evaluate whether the effects were modified by treatment characteristics, we specified based on experiment type and cancer type. We used sensitivity analyses to explore and interpret the sources of high heterogeneity (Higgins JPT and Sterne, 2011). Funnel plots, Begg’s test (Begg and Mazumdar, 1994), and Egger’s test (Egger et al., 1997) would be adopted to detect publication bias only when there are at least 10 studies reporting the primary outcomes, because when there are fewer studies the power of the tests is too low to distinguish chance from real asymmetry (Higgins JPT and Sterne, 2011). Statistical analysis was performed with STATA software, version 13.0 (StataCorp, College Station, TX).

## Results

### Studies Selection

**Figure 1:**
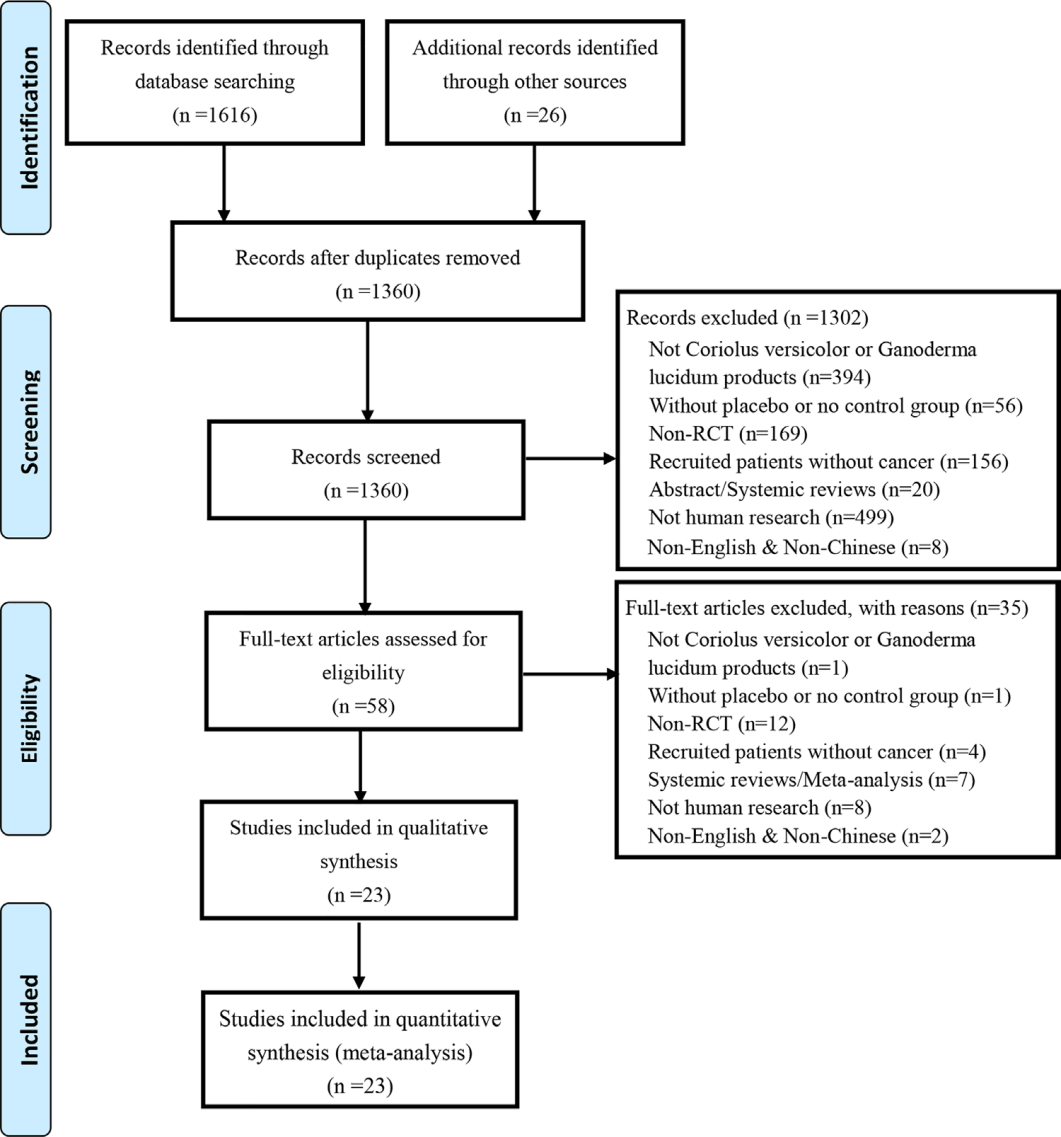
Flow chart.

Our literature search yielded 1,616 trials *via* electronic databases, and 26 trials by hand research. After removing duplicates records, 1,360 trials were screened, and 1,302 trials were excluded by reviewing titles and abstracts. The remaining 58 trials were reviewed by full text. Eventually, 23 trials involving 4,246 cancer patients were included in this work. Study selection flow is detailed by PRISMA flow diagram as shown in **Figure 1**. All the preparations of *C. versicolor* and *G. lucidum* related natural products in each included article were listed in **Supplement 1**.

### Description of Trials Identified

**Table 1** presents the characteristics of the 23 included trials (Niimoto et al., 1988; Go and Chung, 1989; Torisu et al., 1990; Toi et al., 1992; Nakazato et al., 1994; Ogoshi et al., 1995; Morimoto et al., 1996; Shi et al., 1996; Mo et al., 1999; Liang et al., 2002; Tsang et al., 2003; Xu et al., 2003; Jing et al., 2007; Liu et al., 2008; Xu et al., 2008; Zhang et al., 2010; Liu, 2011; Zhao et al., 2012; Hu and GAN, 2013; Zhao et al., 2015; Chay et al., 2017; Miyake et al., 2018; Okuno et al., 2018). All the trials used *C. versicolor* related natural products (n = 14) (Niimoto et al., 1988; Go and Chung, 1989; Torisu et al., 1990; Toi et al., 1992; Nakazato et al., 1994; Ogoshi et al., 1995; Morimoto et al., 1996; Shi et al., 1996; Tsang et al., 2003; Xu et al., 2003; Xu et al., 2008; Chay et al., 2017; Miyake et al., 2018; Okuno et al., 2018) or *G. lucidum* related natural products (n = 9) (Mo et al., 1999; Liang et al., 2002; Jing et al., 2007; Liu et al., 2008; Zhang et al., 2010; Liu, 2011; Zhao et al., 2012; Hu and GAN, 2013; Zhao et al., 2015). The majority of trials were from China (Shi et al., 1996; Mo et al., 1999; Liang et al., 2002; Tsang et al., 2003; Xu et al., 2003; Jing et al., 2007; Liu et al., 2008; Xu et al., 2008; Zhang et al., 2010; Liu, 2011; Zhao et al., 2012; Hu and GAN, 2013; Zhao et al., 2015), whereas eight papers were from Japan (Niimoto et al., 1988; Torisu et al., 1990; Toi et al., 1992; Nakazato et al., 1994; Ogoshi et al., 1995; Morimoto et al., 1996; Miyake et al., 2018; Okuno et al., 2018), one paper was from in Taiwan (Go and Chung, 1989), and one from Singapore (Chay et al., 2017). Almost half of the 23 trials were published in Chinese (Shi et al., 1996; Mo et al., 1999; Liang et al., 2002; Xu et al., 2003; Jing et al., 2007; Liu et al., 2008; Xu et al., 2008; Zhang et al., 2010; Liu, 2011; Hu and GAN, 2013; Zhao et al., 2015), the others were published in English (Niimoto et al., 1988; Go and Chung, 1989; Torisu et al., 1990; Toi et al., 1992; Nakazato et al., 1994; Ogoshi et al., 1995; Morimoto et al., 1996; Tsang et al., 2003; Zhao et al., 2012; Chay et al., 2017; Miyake et al., 2018; Okuno et al., 2018). In 23 trials, 5 included non-small cell lung cancer (NSCLC) patients (Tsang et al., 2003; Liu et al., 2008; Zhang et al., 2010; Hu and GAN, 2013; Zhao et al., 2015), 3 included breast cancer patients (Toi et al., 1992; Morimoto et al., 1996; Kuo et al., 2012; Zhao et al., 2012), 4 included gastric cancer patients (Niimoto et al., 1988; Nakazato et al., 1994; Shi et al., 1996; Xu et al., 2003), 4 included colorectal cancer patients (Torisu et al., 1990; Xu et al., 2008; Liu, 2011; Miyake et al., 2018), 3 included nasopharyngeal carcinoma patients (Go and Chung, 1989; Mo et al., 1999; Liang et al., 2002), and the other 4 trials included esophageal cancer (Ogoshi et al., 1995), rectal cancer (Okuno et al., 2018), gastrointestinal cancer (Jing et al., 2007), and hepatocellular carcinoma patients (Chay et al., 2017), respectively. The range duration of therapy was 1 to 24 months.

**Table 1:** Characteristics of the included trials and participants.

Author	Year	Country	Language	Survey year	Cancer type	TNM stage	KPS	No. of subject	Gender (M/F)	Age median (range)	Experiment	Start time	Daily dosage (/d)	Duration (year)	Control Group	Follow-up (year)	Dropout (T/C)
Zhao et al., 2012	2012	China	English	2009/6–2010/9	Breast cancer	I∼IIIA	NA	48	NA	52.2 (NA)	GL	NA	1g	1/6	RT+CT+placebo	1/12	0/0
Morimoto et al., 1996	1996	Japan	English	1985/2–1988/3	Breast cancer (ER(+))	II	NA	350	NA	52 (28–74)	PSK^a^	2 weeks after surgery	3g	2	CT only	5	8/4
Morimoto et al., 1996	1996	Japan	English	1985/2–1988/3	Breast cancer (ER(+))	II	NA	347	NA	54 (28–75)	PSK^b^	2 weeks after surgery:	3g	2	CT only	5	8/3
Morimoto et al., 1996	1996	Japan	English	1985/2–1988/3	Breast cancer (ER(-))	II	NA	364	NA	52 (27–75)	PSK^c^	2 weeks after surgery:	3g	2	CT only	5	4/8
Toi et al., 1992	1992	Japan	English	1982/10–1985/1	Breast cancer (IIA+T2N1, ER(-))	IIA∼IIIA	NA	278	NA	49.5 (NA)	PSK	2 weeks after surgery:	3g	2	CT only	5	NA
Toi et al., 1992	1992	Japan	English	1982/10–1985/1	Breast cancer (IIIA+T3N0, ER(-))	IIA∼IIIA	NA	81	NA	48.6 (NA)	PSK	2 weeks after surgery:	3g	2	CT only	5	NA
Okuno et al., 2018	2018	Japan	English	2011/10–2013/2	Rectal cancer	II	NA	106	NA	50.1 (NA)	PSK	After surgery	3g	1	Surgery alone	5	2/3
Miyake et al., 2018	2018	Japan	English	2006/3–2010/12	Colorectal cancer	IIB∼III	NA	351	167/184	65.5 (35–80)	PSK	At the same time as chemotherapy	3g	1	CT+leucovorin	5	4/2
Liu, 2011	2011	China	Chinese	2010/5–2011/3	Colorectal cancer	III∼IV	≥70	30	17/13	44 (18–75)	GL	At the same time as chemotherapy	30g	2 cycles	placebo	NA	0/0
Xu et al., 2008	2008	China	Chinese	2003/6–2005/6	Colorectal cancer	III∼IV	>60	53	31/22	61 (41–71)	PSK	NA	6g	1/12	CT only	NA	0/0
Torisu et al., 1990	1990	Japan	English	NA	Colorectal cancer	III∼IV	NA	111	NA	58.9 (34–86)	PSK	10–15 days after surgical operations	3g	1/6	CT+placebo	10	5/4
Ogoshi et al., 1995	1995	Japan	English	NA	Esophageal cancer	I∼IV	NA	69	60/9	61.4 (44–82)	PSK	NA	3g	1/4	RT only	5	0/0
Ogoshi et al., 1995	1995	Japan	English	NA	Esophageal cancer	I∼IV	NA	105	94/11	58.1 (41–77)	PSK	NA	3g	1/4	RT+CT only	5	0/0
Xu et al., 2003	2003	China	Chinese	1997/1–2000/4	Gastric cancer	III	>50	126	78/48	61.3 (36–71)	PSK	2 weeks after surgery	6 grain	2	CT only	5	6/8
Nakazato et al., 1994	1994	Japan	English	1985/7–1987/6	Gastric cancer	I∼IV	NA	253	169/84	58.5 (27–75)	PSK	2 week after surgery	3g	1/6	CT only	7	0/0
Niimoto et al., 1988	1988	Japan	English	NA	Gastric cancer	NA	NA	390	NA	NA (NA)	PSK^d^	1–2 weeks after surgery	3g	1	CT only	5	24/16
Niimoto et al., 1988	1988	Japan	English	NA	Gastric cancer	NA	NA	388	NA	NA (NA)	PSK^e^	1–2 weeks after surgery	3g	1	CT only	5	21/16
Shi et al., 1996	1996	China	Chinese	NA	Gastric cancer	I∼IV	NA	30	NA	NA (NA)	PSP	12–16 days after surgery	3 grain	1/6	CT+Bieganchun	1/6	NA
Jing et al., 2007	2007	China	Chinese	2000/2–2004/12	Gastrointestinal cancer	NA	NA	82	NA	56.3 (NA)	GL^§^	NA	4.5g	1/2	CT only	1/2	0/0
Jing et al., 2007	2007	China	Chinese	2000/2–2004/12	Gastrointestinal cancer	NA	NA	62	NA	56.3 (NA)	GL^¶^	NA	150ml	1/2	CT only	1/2	0/0
Chay et al., 2017	2017	Singapore	English	NA	Hepatocellular carcinoma	NA	NA	15	14/1	61 (48–74)	CV	NA	2.4g	1/2	RT+CT+placebo	NA	0/0
Mo et al., 1999	1999	China	Chinese	NA	Nasopharyngeal carcinoma	I∼IV	≥80	72	67/5	NA (18–60)	GL	At the same time as radiotherapy	2.28g	1/12	RT+Vitamia	1/12	0/0
Liang et al., 2002	2002	China	Chinese	1994/07–1999/01	Nasopharyngeal carcinoma	NA	NA	198	137/61	NA (NA)	GL	At the same time as radiotherapy	1.2g	1/12	RT only	3	1/4
Go and Chung, 1989	1989	Taiwan	English	1981/10–1986/10	Nasopharyngeal carcinoma	I∼IV	≥50	34	26/8	46.5 (NA)	PSK	Within 1 month of the completion of primary treatment	1g	1/12–2	RT only	5	4/0
Zhao et al., 2015	2015	China	Chinese	2011/2–2014/3	NSCLC	III∼IV	≥60	59	40/19	64.1 (39–76)	GL	At the same time as chemotherapy	12 grain	1/2	CT+placebo	NA	0/0
Liu et al., 2008	2008	China	Chinese	2001–2006	NSCLC	NA	>60	56	33/23	45.1 (28–62)	GL	NA	10g	1/4–1/3	CT only	3+1/3	0/0
Zhang et al., 2010	2010	China	Chinese	NA	NSCLC	III∼IV	NA	60	39/21	64.2 (NA)	GL	NA	1 dose	2/3	CT only	NA	0/0
Hu and GAN, 2013	2013	China	Chinese	2011/11–2013/4	NSCLC	III∼IV	≥60	60	38/22	58.8 (18–70)	GL	NA	10g	2/3	CT only	1	0/0
Tsang et al., 2003	2003	China	English	1999/12–2001/4	NSCLC	III∼IV	≥60	68	45/23	58.3 (34–75)	PSP	NA	3 capsules	1/12	CT+placebo	1/12	2/8

PSK, Polysaccharide Krestin; PSP, Polysaccharide Peptide; GL, Ganoderma lucidum; CV, Coriolus versicolor; RT, Radiotherapy; CT, Chemotherapy; NA, not available; NSCLC, Non-small cell lung cancer; M, male; F, female; CT, chemotherapy; RT, radiotherapy; ER(-), estrogen receptor-negative; ER(+), estrogen receptor positive; T, treatment; C, control; *§*, capsule; ¶, oral liquid; a, Stage IIA ER(+), CV+ mitomycin C (MMC) + tamoxifen (TAM) vs. MMC+TAM+ futraful (FT); b, CV+MMC+TAM vs. MMC+TAM; c, Stage IIA ER(-), CV+MMC vs. MMC+FT; d, CV + MMC +FT vs. MMC+FT; e, CV +MMC vs. MMC+FT.

The data in five trials were split into two or three records because there were two kinds of comparison, dosage form or disease staging. The data were split into two records because of the comparison in Ogoshi K’s trial (Ogoshi et al., 1995) [*C. versicolor* + radiotherapy (RT) vs. RT only and *C. versicolor* + RT + chemotherapy (CT) vs. RT + CT only] and in Niimoto M’s trial (Niimoto et al., 1988) [*C. versicolor*+ mitomycin C (MMC)+futraful (FT) vs. MMC+FT and *C. versicolor*+MMC vs. MMC+FT]; because of the dosage form in Junjie Jing’s trial (Jing et al., 2007) (capsule and oral liquid); because of the disease staging in Toi M’s trial (Toi et al., 1992) (stage IIA+T2N1, ER[-], and stage IIIA+T3N0,ER[-]). The data were split into three records because of the comparison and disease staging in Morimoto T’s trial (Morimoto et al., 1996) (stage IIA ER[-]:*C. versicolor*+MMC vs. MMC+FT; stage IIA ER[+]:*C. versicolor*+MMC+ tamoxifen [TAM] vs. MMC+TAM+FT and *C. versicolor*+MMC+TAM vs. MMC+TAM). Therefore, there were 27 records to be analyzed.

**Figures S1** **and** **S2** in the Supplement show the assessment of the risk of bias. All studies were randomized; four studies were double-blinded design; five studies were placebo-controlled trials; six trials described an adequate random sequence generation process; and four trials described the methods used for allocation concealment.

## Effects on Overall Survival

**Figure 2:**
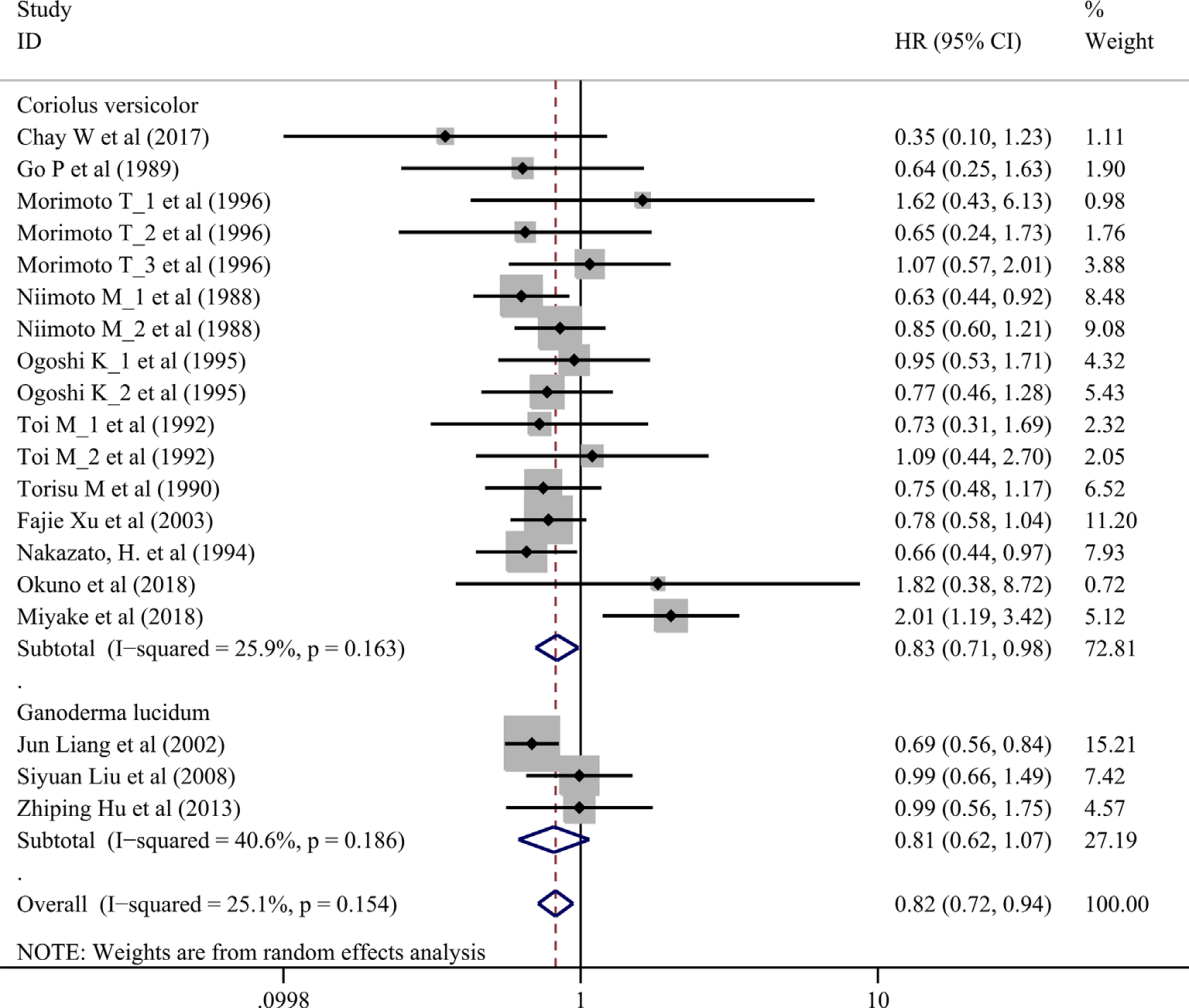
Meta-analysis results of overall survival.

Seventeen trials involving 3,682 cancer patients compared the effect on survival. Compared with control treatment, using of *C. versicolor* and *G. lucidum* related natural products was associated with a lower risk of mortality (*HR*: 0.82 95% *CI*: 0.72, 0.94; *P* = 0.005). Subgroup analysis for experiment type showed this effect was consistent for trial using *C. versicolor* related natural products (*HR*: 0.83; 95% *CI*: 0.71, 0. 98; *P* = 0.030, 16 studies), but no difference was found in trial using *G. lucidum* related natural products (*HR*: 0.81; 95% *CI*: 0.62, 1.07; *P* = 0.139; three studies) (**Figure 2**).

Subgroup analysis for cancer type showed, compared with control treatment, trials using *C. versicolor* and *G. lucidum* related natural products were associated with lower risk of mortality in gastric cancer (*HR*: 0.74; 95% *CI*: 0.62,0.87; *P* = 0.001; four trials) and nasopharyngeal carcinoma (*HR*: 0.68; 95% *CI*: 0.56,0.84; *P* < 0.001; two trials). However, no differences were found in breast cancer (*HR*: 0.95; 95% *CI*: 0.65,1.40; *P* = 0.798; five trials), colorectal cancer (*HR:* 1.22; 95% *CI:* 0.46, 3.21; *P* = 0.694; two trials), esophageal cancer (*HR*: 0.84; 95% *CI*: 0.57,1.24; *P* = 0.387; two trials), hepatocellular carcinoma (*HR*: 0.35; 95% *CI*: 0.10,1.23; *P* = 0.101; one trial), NSCLC (*HR*: 0.99; 95% *CI*: 0.71,1.38; *P* = 0.953; two trials), and rectal cancer (*HR*: 1.82; 95% *CI*: 0.38,8.72; *P* = 0.454; one trial) (**Table 3**).

### Effects on Relapse-Free Survival Rate

**Figure 3:**
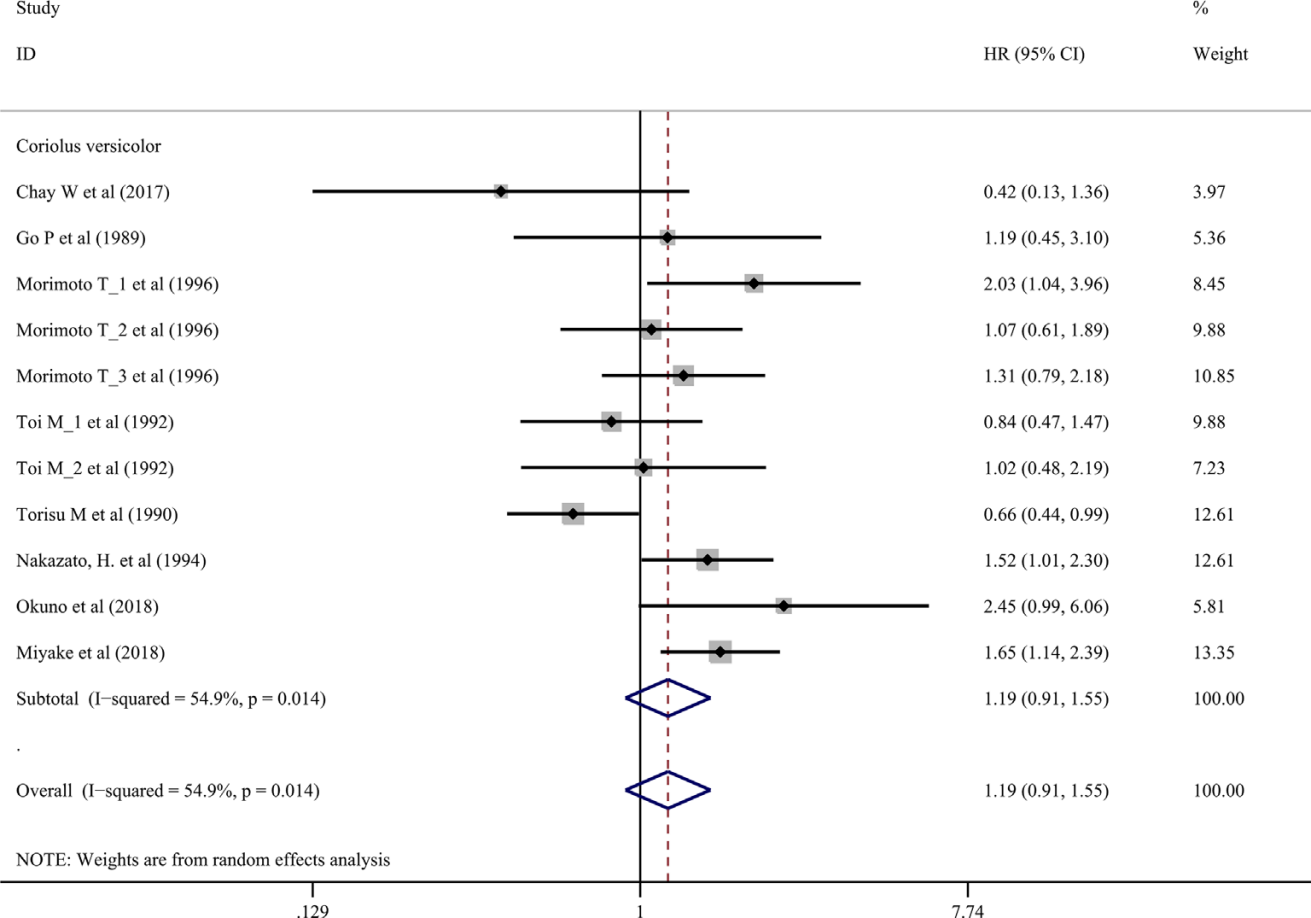
Meta-analysis results of relapse-free survival rate.

Nine trials involving 1,155 cancer patients compared the effect on RFS rate. All nine trials used *C. versicolor* related natural products. As shown in **Figure 3**, there was no significant association of *C. versicolor* related natural products with RFS (*HR*: 1.19; 95% *CI*: 0.91, 1.55; *P* = 0.2) compared with control treatment (**Figure 3**). Subgroup analysis for cancer type showed, compared with control treatment, trials using *C. versicolor* and *G. lucidum* related natural products were associated with higher risk of relapse in gastric cancer (*HR*: 1.52; 95% *CI*: 1.01,2.30; *P* = 0.046; one trial). There were no significant associations of *C. versicolor* related natural products with risk of relapse in breast cancer (*HR*: 1.18; 95% *CI*: 0.89,1.57; *P* = 0.243; five trials), colorectal cancer (HR: 1.05; 95% CI: 0.42,2.58; *P* = 0.923; two trials), hepatocellular carcinoma (*HR*: 0.42; 95% *CI*: 0.13,1.36; *P* = 0.147; one trial), nasopharyngeal carcinoma (*HR*: 1.19; 95% *CI*: 0.45,3.10; *P* = 0.729; one trial), and rectal cancer (HR: 2.45; 95% CI: 0.99,6.06; *P* = 0.053; one trial) (**Table 3**).

### Clinical Efficacy

**Figure 4:**
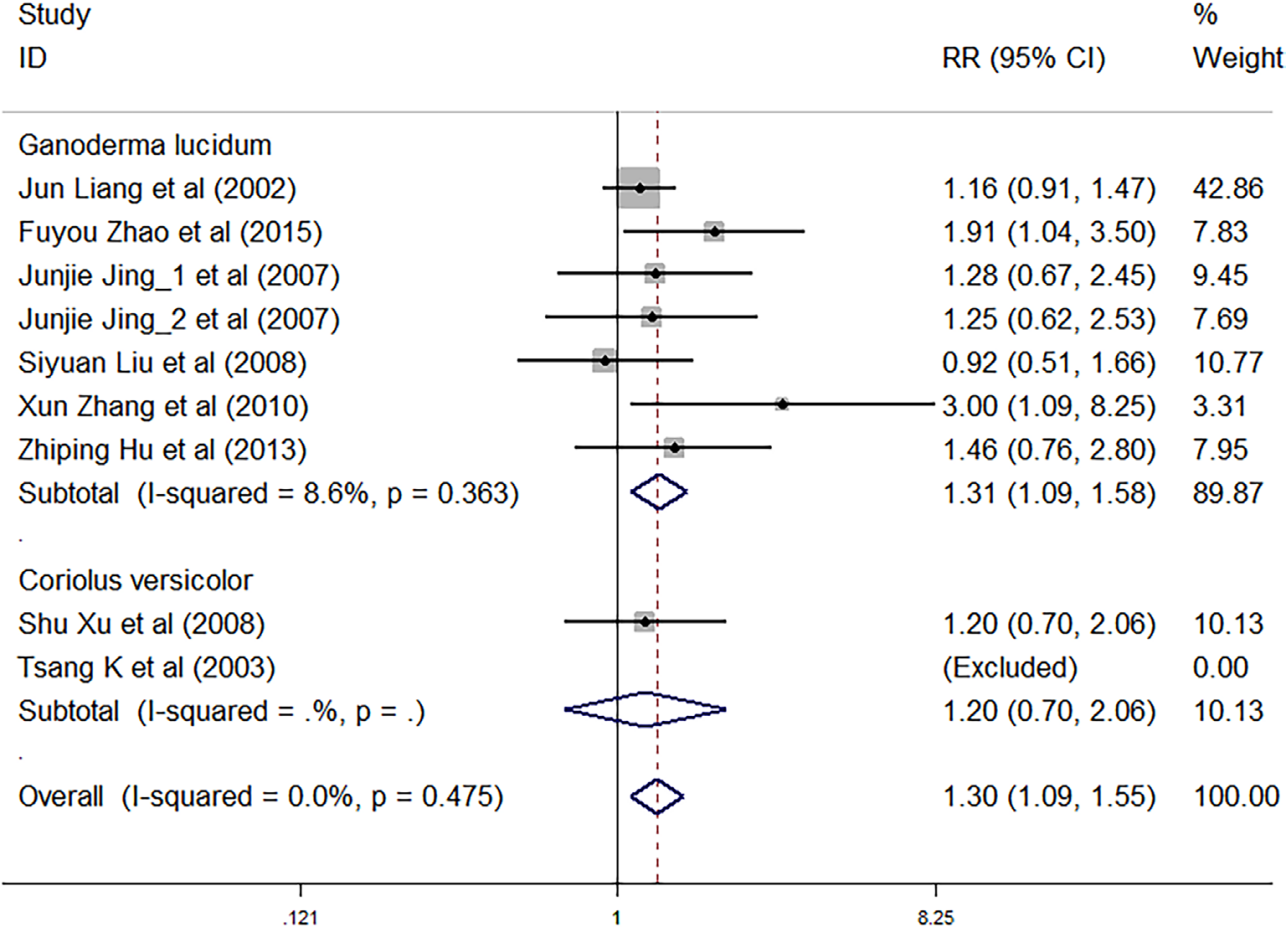
Meta-analysis results of total efficacy.

Nine trials involving 1,883 cancer patients assessed the total efficacy. Compared with control treatment, using *C. versicolor* and *G. lucidum* related natural products was associated with a higher total efficacy (*RR*: 1.30; 95% *CI*: 1.09, 1.55; *P* = 0.003). Subgroup analysis for experiment type showed trials using *G. lucidum* related natural products with a higher total efficacy (*RR*: 1.31; 95% *CI*: 1.09, 1.58; *P* = 0.004; seven studies) compared with control treatment. However, there was no significant association of *C. versicolor* related natural products with total efficacy (*RR*: 1.20; 95% *CI*: 0.70, 2.06; *P* = 0.497) compared with control treatment. Only two trials used *C. versicolor* related natural products, and one of them was excluded because the total efficacy of the trial was 0% (**Figure 4**).

Subgroup analysis for cancer type showed, compared with control treatment, trials using *C. versicolor* and *G. lucidum* related natural products were associated with a higher total efficacy in NSCLC (*RR*: 1.55; 95% *CI*: 1.12, 2.17; *P* = 0.009; five trials), but no differences were found in colorectal cancer (*RR*: 1.20; 95% *CI*: 0.71, 2.06; *P* = 0.497; one trial), gastrointestinal cancer (*RR*: 1.27; 95% *CI*: 0.79,2.04; *P* = 0.329; two trials), and nasopharyngeal carcinoma (*RR*: 1.16; 95% *CI*: 0.91, 1.47; *P* = 0.238; one trial) (**Table 3**).

**Figure 5:**
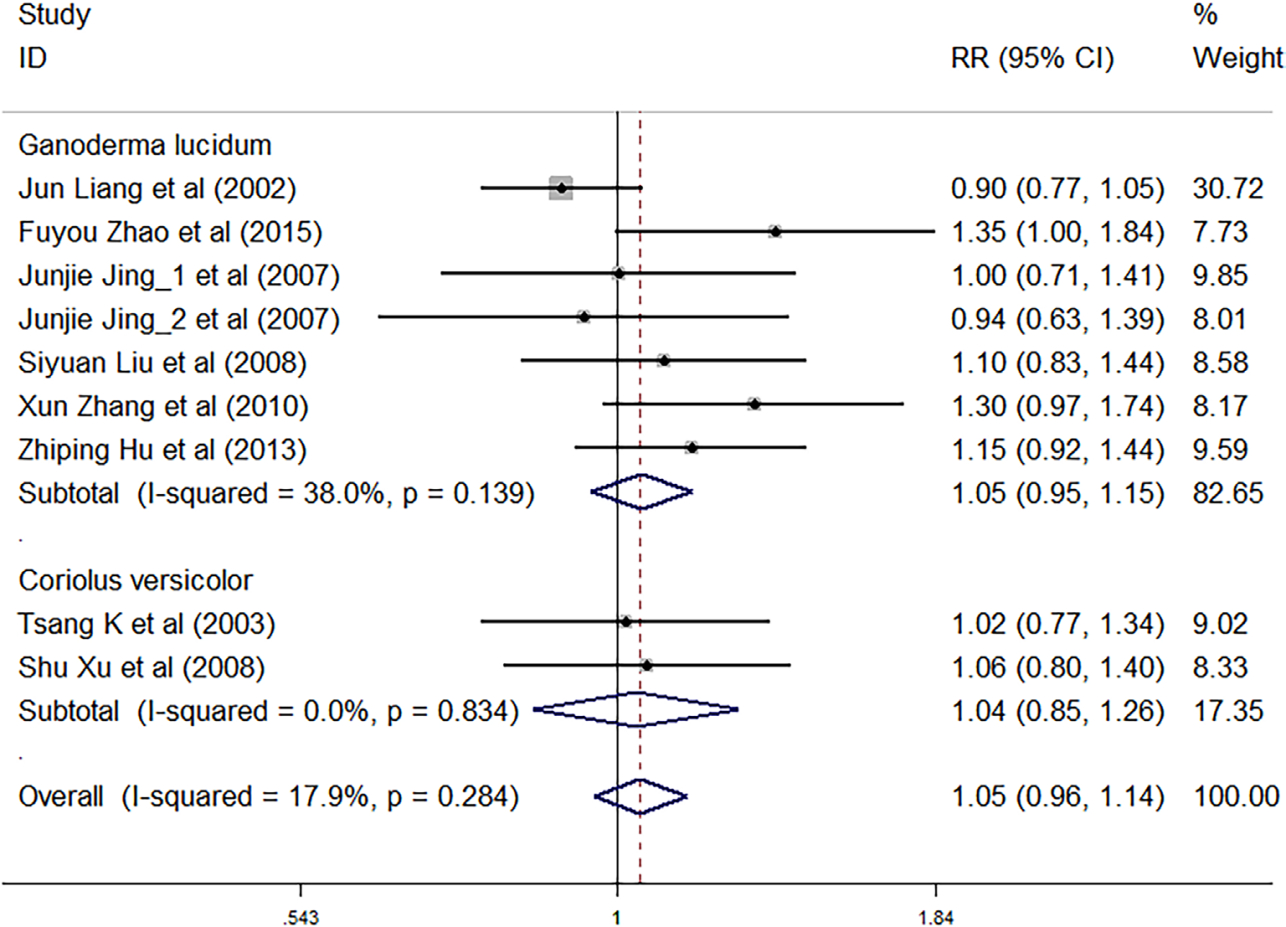
Meta-analysis results of control rate.

Nine trials involving 1,883 cancer patients were compared with the control rate. There was no significant association of *C. versicolor* and *G. lucidum* related natural products and control rate (*RR*: 1.05; 95% *CI*: 0.96, 1.14; *P* = 0.321) compared with control treatment. Subgroup analysis for experiment type showed no significant interactions with experiment type for the primary outcome of control rate. Control rate did not differ significantly between trials using *G. lucidum* related natural products (*RR*: 1.05; 95% *CI*: 0.95, 1.15; *P* = 0.355; seven studies) or trials using *C. versicolor* related natural products (*RR*: 1.04; 95% *CI*: 0.85, 1.26; *P* = 0.725; two studies) and control treatment (**Figure 5**).

Subgroup analysis for cancer type showed, compared with control treatment, trials using *C. versicolor* and *G. lucidum* related natural products were associated with a higher control rate in NSCLC (*RR*: 1.18; 95% *CI*: 1.04, 1.33; *P* = 0.009; five trials), but no differences were found in colorectal cancer (*RR*: 1.06; 95% *CI*: 0.80, 1.40; *P* = 0.684; one trial), gastrointestinal cancer (*RR*: 0.97; 95% *CI*: 0.75,1.26; *P* = 0.836; two trials), and nasopharyngeal carcinoma (*RR*: 0.90; 95% *CI*: 0.77, 1.05; *P* = 0.182; one trial) (**Table 3**).

### Immunomodulating Effects

**Table 2** summarizes results of meta-analysis and subgroup analyses of the immunomodulating effects for *C. versicolor* and *G. lucidum* related natural products. Compared with control treatment, using *C. versicolor* and *G. lucidum* related natural products had a favorable effect on elevated levels of CD3 (*MD*: 9.03%; 95% *CI*: 2.10, 16.50; *P* = 0.011) and CD4 (*MD*: 9.2%; 95% *CI*: 1.01, 17.39; *P* = 0.028). However, no difference between *C. versicolor* and *G. lucidum* related natural products and control treatment was seen in the effect on the levels of CD8 (*MD*: -5.52%; 95% *CI*: -23.17, 12.13; *P* = 0.540) and CD4/CD8 (*MD*: 0.73; 95% *CI*: -0.45, 1.91; *P* = 0.227). In terms of NK, only two trials reported the NK[9, 16]; one trial used *G. lucidum* related natural products in a patient with colorectal cancer (MD: 3.04%; 95% *CI*: -1.76, 7.84; *P* = 0.215), while the other used *C. versicolor* related natural products in a patient with gastric cancer (*MD*: 10.29%; 95% *CI*: 2.07, 18.15; *P* = 0.014); there was no difference between *C. versicolor* and *G. lucidum* related natural products and control treatment in the effect on NK (*MD*: 5.87%; 95% *CI*: -1.06, 12.8; *P* = 0.097) (**Tables 2** and **3**).

**Table 2:** Meta-analysis and subgroup analysis of immunomodulating effects and KPS for *Coriolus versicolor* and *Ganoderma lucidum* related natural products.

Variable	No. of trials	No. of Subject		Heterogeneity		MD/ RR	95% CI	*P* value
		T	C	I^2^	*P* value			
Immunomodulating effects								
CD3 (%)	3	106	109	94.9	<0.001	9.30	(2.10, 16.50)	0.011
*Ganoderma lucidum*	2	46	43	0.0	0.036	13.05	(10.37, 15.72)	<0.001
*Coriolus versicolor*	1	60	66	—	—	4.30	(3.63, 4.97)	<0.001
CD4 (%)	3	106	109	96.4	<0.001	9.20	(1.01,17.39)	0.028
*Ganoderma lucidum*	2	46	43	0.0	0.362	13.19	(10.55, 15.82)	<0.001
*Coriolus versicolor*	1	60	66	—	—	3.12	(2.67, 3.57)	<0.001
CD8 (%)	2	91	94	99.2	<0.001	-5.52	(-23.17, 12.13)	0.540
*Ganoderma lucidum*	1	31	28	—	—	-14.59	(-17.61, -11.57)	<0.001
*Coriolus versicolor*	1	60	66	—	—	3.42	(2.79, 4.05)	<0.001
CD4/CD8	3	106	109	99.7	<0.001	0.73	(-0.45,1.91)	0.227
*Ganoderma lucidum*	2	46	43	93.0	<0.001	1.10	(0.15, 2.04)	0.024
*Coriolus versicolor*	1	60	66	—	—	0.05	(0.04, 0.06)	<0.001
NK (%)	2	30	30	55.1	0.135	5.87	(-1.06,12.80)	0.097
*Ganoderma lucidum*	1	15	15	—	—	3.04	(-1.76, 7.84)	0.215
*Coriolus versicolor*	1	15	15	—	—	10.29	(2.07, 18.51)	0.014
KPS score*								
Effective rate								
*Ganoderma lucidum*	3	75	71	25.7	0.261	1.66	(1.21, 2.26)	<0.001
Stable rate								
*Ganoderma lucidum*	3	75	71	36.8	0.206	1.50	(1.09, 1.16)	0.001

KPS, Karnofsky Performance Status; *, using RR.

**Table 3:** Subgroup analysis of efficacy of the products for cancer type.

Variable	No. of trials	No. of subject		Heterogeneity		MD/ RR/HR	95% *CI*	*P* value
		T	C	I^2^	*P* value			
Overall survival								
Breast cancer	5	706	714	0.0	0.770	0.95	(0.65, 1.40)	0.798
Colorectal cancer	2	230	232	87.2	0.005	1.22	(0.46, 3.21)	0.694
Esophageal cancer	2	94	80	0.0	0.597	0.84	(0.57, 1.24)	0.387
Gastric cancer	4	569	588	0.0	0.620	0.74	(0.62, 0.87)	0.001
Hepatocellular carcinoma*	1	9	6	—	—	0.35	(0.10, 1.23)	0.101
Nasopharyngeal carcinoma*	2	139	93	0.0	0.884	0.68	(0.56, 0.84)	<0.001
NSCLC	2	60	56	0.0	1.000	0.99	(0.71, 1.38)	0.953
Rectal cancer	1	53	53	—	—	1.82	(0.38, 8.72)	0.454
Relapse-free survival rate								
Breast cancer	5	706	714	0.0	0.572	1.18	(0.89, 1.57)	0.243
Colorectal cancer	2	230	232	90.5	0.001	1.05	(0.42, 2.58)	0.923
Gastric cancer*	1	129	124	—	—	1.52	(1.01, 2.30)	0.046
Hepatocellular carcinoma	1	9	6	—	—	0.42	(0.13, 1.36)	0.147
Nasopharyngeal carcinoma	1	17	17	—	—	1.19	(0.45, 3.10)	0.729
Rectal cancer	1	53	53	—	—	2.45	(0.99, 6.06)	0.053
Clinical efficacy								
Total efficacy								
Colorectal cancer	1	27	26	—	—	1.20	(0.71, 2.06)	0.497
Gastrointestinal cancer	2	84	60	0.0	0.959	1.27	(0.79, 2.04)	0.329
Nasopharyngeal carcinoma	1	122	76	—	—	1.16	(0.91, 1.47)	0.238
NSCLC*	5	121	114	41.8	0.161	1.55	(1.12, 2.17)	0.009
Control rate								
Colorectal cancer	1	27	26	—	—	1.06	(0.80, 1.40)	0.684
Gastrointestinal cancer	2	84	60	0.0	0.803	0.97	(0.75, 1.26)	0.836
Nasopharyngeal carcinoma	1	122	76	—	—	0.90	(0.77, 1.05)	0.182
NSCLC*	5	155	148	0.0	0.623	1.18	(1.04, 1.33)	0.009
Immunomodulating effects								
CD3								
Colorectal cancer	1	15	15	—	—	10.44	(4.22, 16.66)	0.001
Gastric cancer	1	60	66	—	—	4.30	(3.63, 4.97)	<0.001
NSCLC	1	31	28	—	—	13.64	(10.67, 16.61)	<0.001
CD4*								
Colorectal cancer	1	15	15	—	—	11.05	(5.75, 16.35)	<0.001
Gastric cancer	1	60	66	—	—	3.12	(2.67, 3.57)	<0.001
NSCLC	1	31	28	—	—	13.89	(10.85, 16.93)	<0.001
CD8*								
Gastric cancer	1	60	66	—	—	3.42	(2.79, 4.05)	<0.001
NSCLC	1	31	28	—	—	-14.59	(-17.61, -11.57)	<0.001
CD4/CD8*								
Colorectal cancer	1	15	15	—	—	0.58	(0.09, 1.07)	0.020
Gastric cancer	1	60	66	—	—	0.05	(0.04, 0.06)	<0.001
NSCLC	1	31	28	—	—	1.55	(1.44, 1.67)	<0.001
NK								
Colorectal cancer	1	15	15	—	—	3.04	(-1.76, 7.84)	0.215
Gastric cancer*	1	15	15	—	—	10.29	(2.07, 18.51)	0.014
KPS score*								
Effective rate								
Colorectal cancer	1	15	15	—	—	2.00	(0.76, 5.24)	0.159
NSCLC	2	60	56	54.1	0.140	1.60	(1.16, 2.21)	0.004
Stable rate								
Colorectal cancer	1	15	15	—	—	1.30	(0.86, 1.96)	0.209
NSCLC*	2	60	56	60.4	0.112	1.62	(1.27, 2.04)	<0.001

NSCLC, Non-small cell lung cancer. *P < 0.05 means statistically significant.

Subgroup analysis for experiment type of CD3 showed the favorable effect on elevating the levels of CD3 both for trials using *G. lucidum* related natural products (*MD*: 13.05%; 95% *CI*: 10.37, 15.72; *P* < 0.001; two trials) and *C. versicolor* related natural products (*MD*: 4.30%; 95% *CI*: 3.63, 4.97; *P* < 0.001; one trials) compared with control group. The subgroup analysis of CD4 was consistent with CD3; trials using *G. lucidum* related natural products (*MD*: 13.19%; 95% *CI*: 10.55, 15.82; *P* < 0.001; two trials) or *C. versicolor* related natural products (MD: 3.12%; 95% *CI*: 2.67, 3.57; *P* < 0.001; one trials) had favorable effect on elevating the levels of CD4 (**Table 2**).

Subgroup analysis for experiment type of CD8 showed the favorable effect on felling the level of CD8 both for one trial using *G. lucidum* related natural products (MD: -14.59%; 95% *CI*: -17.61, -11.57; *P* < 0.001) and one trial using *C. versicolor* related natural products (*MD*: 3.42%; 95% *CI*: 2.79, 4.05; *P* < 0.001) compared with the control group. Subgroup analysis of CD4/CD8 showed the favorable effect on elevating the levels of CD4/CD8 both for trials using *G. lucidum* related natural products (*MD*: 1.1 95% *CI*: 0.15, 2.04; *P* = 0.024; two trials) and *C. versicolor* related natural products (*MD*: 0.05; 95% *CI*: 0.04, 0.06; *P* < 0.001; one trials) compared with the control group (**Table 2**).

**Table 3** summarizes results of subgroup analyses for cancer type and immunomodulating effects. There was one trial that reported that *C. versicolor* and *G. lucidum* related natural products had a favorable effect on elevating the levels of CD3, CD4, and CD4/CD8 in colorectal cancer, gastric cancer, and NSCLC, respectively. One trial in gastric cancer reported that the products had a favorable effect on elevating the levels of CD8, but one trial in NSCLC reported that the products had an effect on felling the levels of CD8 (**Table 3**).

### Post-Treatment KPS Score Change

Three trials using *G. lucidum* related natural products were compared with the post-treatment KPS score change. Compared with the control treatment, using *G. lucidum* related natural products was associated with a higher total efficacy (*RR*: 1.66; 95% *CI*: 1.21, 2.26; *P* < 0.001) and higher stable rate (*RR*: 1.50; 95% *CI*: 1.09, 1.16; *P* = 0.001) (**Table 2**). Subgroup analyses for cancer type showed, compared with control treatment, that using *G. lucidum* related natural products was associated with a higher total efficacy (*RR*: 1.60; 95% *CI*: 1.16, 2.21; *P* = 0.004; two trials) and higher stable rate (*RR*: 1.62; 95% *CI*: 1.27, 2.04; *P* < 0.001; two trials) in NSCLC. However, one trial reported the association between *G. lucidum* related natural products and post-treatment KPS score change in colorectal cancer (**Table 3**).

### Adverse Events

In all 23 trials, one trial in gastric cancer mentioned the severe adverse events (Shi et al., 1996); seven trials (two in NSCLC; one in breast cancer; one in nasopharyngeal carcinoma; one in gastric cancer; two in colorectal cancer) reported that adverse effects fell by using *C. versicolor* and *G. lucidum* related natural products (Morimoto et al., 1996; Liang et al., 2002; Xu et al., 2003; Xu et al., 2008; Zhang et al., 2010; Zhao et al., 2015; Miyake et al., 2018). The most common adverse effects happened in including nausea or/and vomiting, leucopenia, diarrhea, thrombocytopenia, liver dysfunction, general fatigue, and anorexia.

### Publication Bias and Sensitivity Analyses

**Figure 6:**
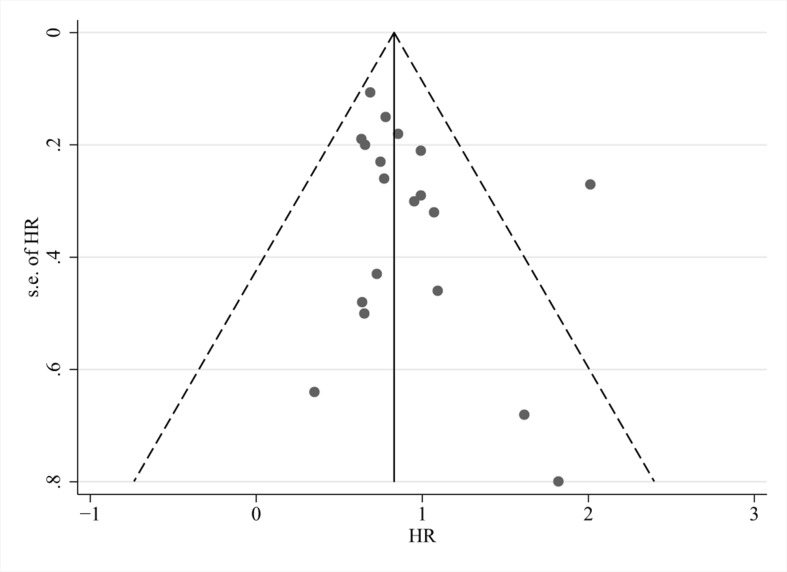
Funnel plots of overall survival.

Visual inspection of funnel plots (**Figure 6**), Begg’s test (*P* = 0.294), and Egger’s test (*P* = 0.162) revealed no evidence of publication bias for the examined primary outcomes. We did sensitivity analyses by excluding four trials using the decoction (Liu et al., 2008; Zhang et al., 2010; Liu, 2011; Hu and GAN, 2013); effects on OS (*HR*: 0.81; 95% *CI*: 0.69, 0.93; *P* = 0.004), total efficacy (*RR*: 1.26; 95% *CI*: 1.04, 1.53; *P* = 0.018), and control rate (*RR*: 1.00; 95% *CI*: 0.90, 1.11; *P* = 0.957) showed that the results did not change.

## Discussion

To the best of our knowledge, this study is the first meta-analysis focusing on *C. versicolor* and *G. lucidum* related natural products as adjuvant treatment on cancer patients. The pooled analysis demonstrated that *C. versicolor* and *G. lucidum* related natural products were significantly associated with lower risks of mortality in patients with cancers, and the pooled *HR* of patient in both groups is 0.82. Moreover, the effect on RFS is only available for *C. versicolor* related trials and results no significant difference across the patients. The side effect profiles show that these products were well tolerated. As a result, from aspects of clinical efficacy and safety, this study suggested that both *C. versicolor* and *G. lucidum* related therapy can be considered as an additional treatment option over different stages and types of cancers, although this recommendation cannot be specifically conclusive because the review only included limited kinds and stages of cancers.

With more evidence proving the anticancer effect of *C. versicolor* and *G. lucidum* extracts, lack of guidelines to support the clinical use for patients with cancers gradually becomes an issue. Especially in Asia, several companies have been dedicated to prepare anti-cancer formulae from *C. versicolor* and *G. lucidum* extracts using new technology (Patel and Goyal, 2012). As the companies put huge amount of resources in marketing, as supplements, there is a booming demand of *C. versicolor* and *G. lucidum* related natural products for preventing and treating of cancers, and thus more companies are expected to participate in the potential market. However, based on the amount of clinical trials found in this review study, it is necessary to accelerate human trials of *C. versicolor* and *G. lucidum* extracts to verify their efficacy and safety in various types and stages of cancers. For cancer patients and their families, clinical evidence and guidelines recommending *C. versicolor* and *G. lucidum* related natural products as an additional treatment with conventional cancer therapies are critical to improve the survival chance. In this study, meta-analysis on the immunomodulating effects also showed that both *C. versicolor* and *G. lucidum* extracts can significantly elevate the levels of CD3 and CD4 T cell. The CD3 and CD4 T cell count alongside other immunological parameters are critical in monitoring immune function, and the CD4 T cell subset is used as a standard for assessing the progression of disease. Lower levels of CD3 and CD4 T cell are related to immunosuppression of chemotherapy or radiotherapy (Tsegaye et al., 1999). The results of increasing the levels of CD3 and CD4 indicated that these products can help to reduce the immunosuppression of chemotherapy or radiotherapy.

The anticancer values of *C. versicolor* and *G. lucidum* have progressed for decades since the analysis of their extracts. Regarding the fact that clinicians usually confuse *C. versicolor* and *G. lucidum* since their dried herbs have similar appearance and nature of medicinals, this review has demonstrated distinguishable benefit of *C. versicolor* and *G. lucidum* extracts in different types and stages of cancer therapies as supplements. However, one of the best ways to prevent confusion is to promote proprietary Chinese medicine, for instance, capsule, pill, or dissolvable granule, and develop regular drugs for regions outside of East Asia. Learnt from this review, the future direction of *C. versicolor* and *G. lucidum* extract trials can be aimed to compare efficacy based on different standard dosages, or their independent therapeutic effect on patients with end stage cancers.

Several limitations are encountered during this study. First, the number of clinical trials is limited and mainly conducted in Asia. There is a lack of a large number of patients with the same type and stage of cancer, and the generalizability is limited. Second, the *C. versicolor* and *G. lucidum* extracts in trials are prepared differently and with various dosages. Therefore, we could not effectively examine the dosage effect of treatment across the outcomes. Third, although the adverse events (AE)/serious adverse events (SAE) profile is an important factor for choosing treatment options, it was not possible to perform an analysis to deal with such a concern because AE/SAE are not fully reported in all included trials. Fourth, we lack the dose-response analysis of these products because most of the original data did not mention the dose response; we could not conduct the dose-response analysis. In addition, as some patients are undergoing post-surgery and/or radiotherapy or chemotherapy, causation between the events and *C. versicolor* and *G. lucidum* extracts is hard to be evaluated. Fourth, in the included trials, *C. versicolor* and *G. lucidum* extracts can be independently applied or combined with other drugs as interventions; therefore, some of the therapeutic effect can be due to the interacted result between *C. versicolor* and *G. lucidum* extracts and other components.

## Conclusion

In this meta-analysis, we found that *C. versicolor* and *G. lucidum* related natural products could increase the OS in cancer patients. Besides, it seems likely that the products provide clinical and life quality benefits for cancer patients with low side effects. Large sample size and high-quality randomized controlled trial (RCT) in different continents with various types and stages of cancer are needed to further evaluate the effect of the products on patients in the future.

## Author Contributions

ZB designed and supervised this study. LZ, PY, and WL wrote the manuscript. LZ and PY searched the data and extracted the data. LY and PY provided the searching strategy and data analysis. PY and WL assessed the risk of bias. All authors read and approved the final manuscript.

## Funding

The present work was supported by a grant from the Innovation and Technology Fund from Hong Kong Innovation and Technology Commission (ITS/134/15FX).

## Conflict of Interest Statement

The authors declare that the research was conducted in the absence of any commercial or financial relationships that could be construed as a potential conflict of interest.

The handling editor declared a shared affiliation, though no other collaboration, with the authors at the time of review.

## Supplementary Material

Figure S1 Number/proportions of trials that met each criterion for risk of bias.

Figure S2 Results of the risk of bias for 23 included trials.

Table S1 The preparation of Coriolus versicolor and Ganoderma lucidum related natural products in each included articles.
