# Supplementary figures and images for: Coriolus Versicolor and Ganoderma Lucidum Related Natural Products as an Adjunct Therapy for Cancers: A Systematic Review and Meta-Analysis of Randomized Controlled Trials
Source: Front Pharmacol. 2019 Jul 3;10:703. doi: 10.3389/fphar.2019.00703 (PMC6616310; doi:10.3389/fphar.2019.00703)

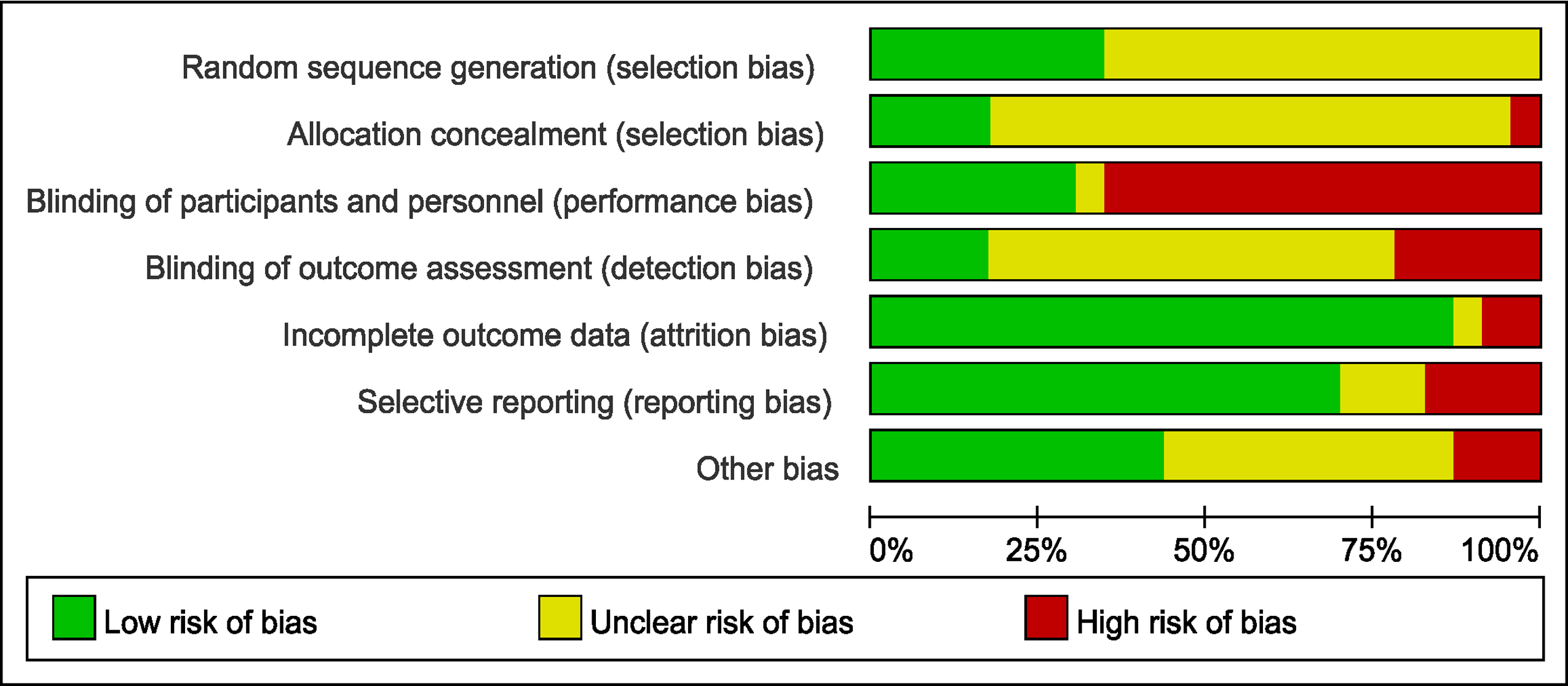

Supplement: Figure S1 — Number/proportions of trials that met each criterion for risk of bias. [file Image_1.tif]

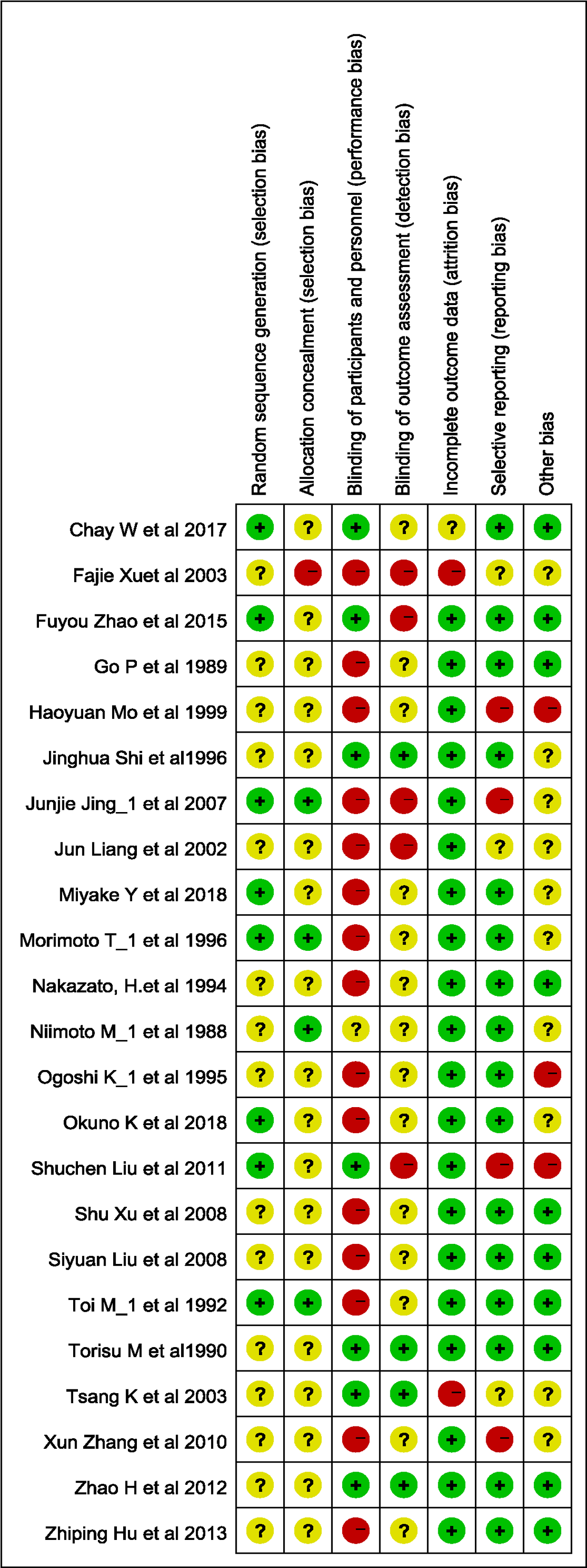

Supplement: Figure S2 — Results of the risk of bias for 23 included trials. [file Image_2.tif]
