# Supplementary material for: Coriolus Versicolor and Ganoderma Lucidum Related Natural Products as an Adjunct Therapy for Cancers: A Systematic Review and Meta-Analysis of Randomized Controlled Trials
Source: Front Pharmacol. 2019 Jul 3;10:703. doi: 10.3389/fphar.2019.00703 (PMC6616310; doi:10.3389/fphar.2019.00703)
Supplement: Table S1 — The preparation of Coriolus versicolor and Ganoderma lucidum related natural products in each included articles. [file Table_1.docx]

**Supplement 1** The preparation of Coriolus versicolor and Ganoderma lucidum related natural products in each included articles

| Author | Year | Country | Language | Experiment | Daily Dosage (/d) | Composition and Quality Control |
| --- | --- | --- | --- | --- | --- | --- |
| Zhao H et al | 2012 | China | English | GL | 1g | The spore powder of Ganoderma lucidum was sourced from the dry sporephore of Ganoderma lucidum (Leyss. ex Fr.) Karst and processed according to the follow protocol through removing from foreign matter and drying in the shade. The processing followed the Chinese Pharmacopoeia 2010. The authenticity of G lucidum was verified prior to the experiments. Quality control was reported. A chemical analysis of the material was not included. |
| Morimoto T et al | 1996 | Japan | English | PSK | 3g | PSK is a protein-bound polysaccharide derived from the mycelium of a Basidiomycetes, Coriolus versicolor. The spore power of Coriolus versicolor was sourced from the dry sporephore of Coriolus versicolor (L. ex Fr.) Quel and processed according to the follow protocal through removing from foreign matter and drying in the sun. The processing followed the Japanese Pharmacopoeia 1996. The authenticity of C versicolor was verified prior to the experiments. Quality control was reported. A chemical analysis of the material was not included. |
| Toi M et al | 1992 | Japan | English | PSK | 3g | PSK is a protein-bound polysaccharide extracted from the mycelium of Coriolus versicolor. The processing followed the Japanese Pharmacopoeia 1991. The authenticity of C versicolor was verified prior to the experiments. Quality control was reported. A chemical analysis of the material was not included. |
| Shuchen Liu et al | 2011 | China | Chinese | GL | 30g | Yiqiyangyin granules were provided by Fujian university of traditional Chinese medicine and manufactured by Guangxi pili pharmaceutical factory production.The composition of granules included Astragali Radix (30g), Ganoderma (30g), Ligustri Lucidi Fructus (15g) and Dioscoreae Rhizoma (15g). The batch numbers were A090260-01 and A090768-01. The spore powder of Ganoderma lucidum was sourced from the dry sporephore of Ganoderma lucidum (Leyss. ex Fr.) Karst and processed according to the follow protocol through removing from foreign matter and drying in the shade. The processing followed the Chinese Pharmacopoeia 2010. The authenticity of G lucidum was verified prior to the experiments. Quality control was not reported. A chemical analysis of the material was not included. |
| Shu Xu et al | 2008 | China | Chinese | PSK | 6g | PSK is a protein-bound polysaccharide extracted from the mycelium of Coriolus versicolor. The processing followed the Chinese Pharmacopoeia 2005. The authenticity of Coriolus versicolor was verified prior to the experiments. Quality control was not reported. A chemical analysis of the material was not included. |
| Torisu M et al | 1990 | Japan | English | PSK | 3g | polysaccharide K(PSK),a plant-derived polysaccharide, A boiled aqueous extract of the cultured mycelia of Coriolus vesiocolor, a Basidiomycetes fungus,was precipitated by ammonium sulfate and the desalted powder was designated as PSK(protein-bound polysaccharide Kreha). The processing followed the Japanese Pharmacopoeia 1986. The authenticity of C versicolor was verified prior to the experiments. Quality control was reported. A chemical analysis of the material was not included. |
| Ogoshi K et al | 1995 | Japan | English | PSK | 3g | Protein-bound plosysoccharide K(PSK：Krestin.Kureha Chemical Industry .Tokyo.Japan) is an anti-tumo drug parpred from Coliolous Vesicolor（Fr）Quel a member of the Basidiomycetes.It inclouds both protein and ploysaccharide components and has a melocular weight of approximately 100,000. The processing followed the Japanese Pharmacopoeia 1991. The authenticity of C versicolor was verified prior to the experiments. Quality control was reported. A chemical analysis of the material was not included. |
| Fajie Xu et al | 2003 | China | Chinese | PSK | 6grain | Polysaccharide capsules is made of a protein-bound polysaccharide extracted from mycelia of Coriolus ersicolor and the main ingredient are (1→3)-β-D-glucan and (1→6)-β-D-glucan. The processing followed the Chinese Pharmacopoeia 2000. The authenticity of Coriolus versicolor was verified prior to the experiments. Quality control was not reported. A chemical analysis of the material was not included. |
| Nakazato, H. et al | 1994 | Japan | English | PSK | 3g | PSK（Kureha Chemical Industry Co Ltd, Japan) is a protein-bound polysaccharide ((β-D-glucan) extracted from mycelia of Coriolus ersicolor (strain CM-101) of the Basidiomycetes. The processing followed the Japanese Pharmacopoeia 1991. The authenticity of C versicolor was verified prior to the experiments. Quality control was reported. A chemical analysis of the material was not included. |
| Niimoto M et al | 1988 | Japan | English | PSK | 3g | PSK is a protein bound polysaccharide derived from Coriolus Versicolor, belonging to the Polyporus of Basidiomycetes and is defined as a host mediated non-specific immunomodulator. The processing followed the Japanese Pharmacopoeia 1986. The authenticity of C versicolor was verified prior to the experiments. Quality control was reported. A chemical analysis of the material was not included. |
| Jinghua Shi et al | 1996 | China | Chinese | PSP | 3grain | Polysaccharopeptide (PSP) is provided by the qingkang pharmaceutical factory of Shanghai normal university and is extracted from the mycelium of Coriolus Versicolo (strain COV-1). The processing followed the Chinese Pharmacopoeia 1995. The authenticity of Coriolus versicolor was verified prior to the experiments. Quality control was not reported. A chemical analysis of the material was not included. |
| Junjie Jing et al | 2007 | China | Chinese | GL | 150ml | The composition of Bailong capsule and mixture is Ginseng Radix et Rhizoma, Ganoderma, Agrimonia Herba, Hirudo, etc. Bailong capsule is prepared by the same experience prescription of clinical application in Shanxi Provincial Cancer Hospital for long-term clinical application and reliable efficacy.The processing followed the Chinese Pharmacopoeia 2005. The authenticity of G lucidum was verified prior to the experiments. Quality control was reported. A chemical analysis of the material was included. |
| Chay W et al | 2017 | Singapore | English | CV | 2.4g | Coriolus versicolor (CV), a fungus of the Basidiomycetes family; Polysaccharide peptide (PSP) isolated from CV. The authenticity of Coriolus versicolor was verified prior to the experiments. Quality control was not reported. A chemical analysis of the material was not included. |
| Haoyuan Mo et al | 1999 | China | Chinese | GL | 2.28g | Five-color Ganoderma capsule is composed of Ganoderma, Poria, and so on, provided by Hong Kong waijian health products co., LTD., and manufactured by guangxi weijian pharmaceutical co., LTD. Five-color Ganoderma lucidum has a positive effect on helping to kill tumor cells and improve their own immune function. The processing followed the Chinese Pharmacopoeia 1995. The authenticity of Ganoderma lucidum was verified prior to the experiments. Quality control was not reported. A chemical analysis of the material was not included. |
| Jun Liang et al | 2002 | China | Chinese | GL | 1.2g | The composition of Lingzhi-912 capsule is Pheretima and Ganoderma. Ganoderma lucidum-912 has significantly stronger anti-tumor and immune-enhancing effects than 912. The processing followed the Chinese Pharmacopoeia 2000. The authenticity of Ganoderma lucidum was verified prior to the experiments. Quality control was not reported. A chemical analysis of the material was not included. |
| Go P et al | 1989 | Taiwan | English | PSK | 1g | PSK polysaccharide (Krestin'") derivative is a protein-bound from the mycelium of Coriolus versicolor, a Basidiomycetes fungus. The processing followed the Chinese Pharmacopoeia 1985. The authenticity of Coriolus versicolor was verified prior to the experiments. Quality control was not reported. A chemical analysis of the material was not included. |
| Fuyou Zhao et al | 2015 | China | Chinese | GL | 12grain | Resolving Toxin Prescription（Including LucidGanoderma，Seed of Jobstears，Ornithogalum umbellatum）.The main composition of Fufang Lingzhibaozi capsule is Ganoderma spore. Ganoderma lucidum polysaccharide has obvious inhibitory effect on tumor cells. The processing followed the Chinese Pharmacopoeia 2015. The authenticity of Ganoderma lucidum was verified prior to the experiments. Quality control was not reported. A chemical analysis of the material was not included. |
| Siyuan Liu et al | 2008 | China | Chinese | GL | 10g | The prescription composition of Zhongliu formula is Ginseng Radix et Rhizoma (15g), Poria (15g), Bombyx Batryticatus (15g), Trichosanthis Pericarpium (15g), Lysimachiae Herba (15g), Pheretima (15g), Ligustri Lucidi Fructu (15g), Paridis Rhizoma (15g), Atractylodis Macrocephalae Rhizoma (10g), Arisaematis Rhizoma (10g), Aurantii Fructus (10g), Common Camptotheca Fructu (10g), Ganoderma (15g), Citri Reticulatae Pericarpium (10g), Pinelliae Rhizoma Praeparatum (10g), Astragali Radix (30g), Mylabris (0.1g), Glycyrrhizae Radix et Rhizoma (5g). Ganoderma lucidum after decoction is used as a medicine. The processing followed the Chinese Pharmacopoeia 2005. The authenticity of Ganoderma lucidum was verified prior to the experiments. Quality control was not reported. A chemical analysis of the material was not included. |
| Xun Zhang et al | 2010 | China | Chinese | GL | 1dose | The composition of Resolving Toxin Prescription is Ganoderma, Coicis Semen, Ornithogalum caudatum and so on. The processing followed the Chinese Pharmacopoeia 2010. The authenticity of Ganoderma lucidum was verified prior to the experiments. Quality control was not reported. A chemical analysis of the material was not included. |
| Zhiping Hu et al | 2013 | China | Chinese | GL | 10g | The main ingredient of Bailonglingshatang is Solanum lyratum (30g), Solanum nigrum (30g), Ganoderma (10g), Glehniae Radix (15g)，studies have shown that GL polysaccharide, an anti-tumor component in GL, has a broad-spectrum anti-tumor effect and enhances immunity. The processing followed the Chinese Pharmacopoeia 2010. The authenticity of Ganoderma lucidum was verified prior to the experiments. Quality control was not reported. A chemical analysis of the material was not included. |
| Tsang K et al | 2003 | China | English | PSP | 3capsules | polysaccharide peptides (PSP) (Windsor Pharmaceutics,Hong Kong),PSP has a molecular weight of 100k and is prepared from cultured mycelium of the COV-1 strain of Coriolus versicolor.A group of polysaccharide peptides (PSP) has been isolated from the mycelium of Yun-zhi. The processing followed the Chinese Pharmacopoeia 2000. The authenticity of Coriolus versicolor was verified prior to the experiments. Quality control was not reported. A chemical analysis of the material was not included. |
| Yasuhiro Miyake et al. | 2017 | Japan | English | PSK | 3.0g | Protein-bound polysaccharide K (PSK) is isolated and purified from the cultured mycelium of Basidiomycete Coriolus versicolor. A group of polysaccharide peptides (PSP) has been isolated from the mycelium of Yun-zhi. The processing followed the Chinese Pharmacopoeia 2010. The authenticity of Coriolus versicolor was verified prior to the experiments. Quality control was not reported. A chemical analysis of the material was not included. |
| Kiyotaka Okuno et al. | 2017 | Japan | English | PSK | 3.0 | Protein-bound polysaccharide K (KRESTIN, PSK; Kureha Chemical Industry Co., Tokyo, Japan) is extracted from the mycelia of Coriolus versicolor. Quality control was not reported. A chemical analysis of the material was not included. The processing followed the Chinese Pharmacopoeia 2010. The authenticity of Coriolus versicolor was verified prior to the experiments. |

PSK: Polysaccharide Krestin; PSP: Polysaccharide Peptide; GL: Ganoderma lucidum; CV: Coriolus versicolor.
